# Severe Hypocalcemia and Dramatic Increase in Parathyroid Hormone after Denosumab in a Dialysis Patient: A Case Report and Review of the Literature

**DOI:** 10.1155/2019/3027419

**Published:** 2019-03-21

**Authors:** Ravinder D. Bhanot, Jasleen Kaur, Zeenat Bhat

**Affiliations:** ^1^Division of Pulmonary and Critical Care, St. Mary's of Michigan Medical Center, Saginaw, MI, USA; ^2^Department of Internal Medicine, Wayne State University, Detroit, MI, USA; ^3^Division of Nephrology, Department of Internal Medicine, Wayne State University, Detroit, MI, USA

## Abstract

Chronic kidney disease-mineral and bone disorder (CKD-MBD) is frequently present in advanced stages of chronic kidney disease (CKD) patients with high risk of fracture and elevated socioeconomic burden. Denosumab, an injectable human monoclonal antibody with affinity for nuclear factor-kappa ligand (RANKL), is an effective treatment for osteoporosis in postmenopausal women and men. Unlike the bisphosphonates, the pharmacokinetics and pharmacodynamics of denosumab are not influenced by the renal function and are being increasingly used for patients having CKD-MBD with low bone mineral density (BMD) to reduce the risk of fragility fractures. Hypocalcemia is a known side effect of this drug along with compensatory increase in parathyroid hormone (PTH). However, limited information is available in the literature regarding this potentially life-threatening side effect with denosumab in end-stage renal disease (ESRD) patients on dialysis. We present a patient with ESRD on peritoneal dialysis who developed severe symptomatic hypocalcemia and dramatic increase in PTH following denosumab therapy. She was conservatively managed with calcium supplementation and appropriate adjustment in calcium dialysate. We have also reviewed the literature on the use of denosumab in dialysis patients and looked at additional factors that may precipitate severe hypocalcemia in these patients. We believe that denosumab should be used with caution in dialysis patients since it may lead to profound hypocalcemia. Clinicians should ensure special attention in recognizing patients at risk of developing this serious adverse effect, so that prompt treatment and preventive strategies can be implemented.

## 1. Introduction

Disorders of mineral and bone metabolism are common sequelae of chronic kidney disease (CKD) that are collectively termed as chronic kidney disease-mineral and bone disorder (CKD-MBD). It often leads to increased bone fragility and fractures, due to varying combinations of low bone mineral content and abnormal bone quality. More severe stages of CKD (stages 4–5D) are associated with progressively reduced bone mineral density (BMD), a higher prevalence of fracture [1], and a mortality rate after fracture about twice as high as that compared to those without severe CKD [2].

Denosumab is a fully human monoclonal antibody that specifically binds to the receptor activator of nuclear factor-*κ*B ligand (RANKL) [3] leading to reduced osteoclast activity and bone resorption. When compared with bisphosphonates, it has greater effectiveness in increasing bone mineral density (BMD) [4] and better pharmacologic profile especially in CKD patients and is being increasingly used for patients having CKD-MBD with low BMD to reduce the risk of fragility fractures. It is known to cause severe hypocalcemia in certain high-risk individuals along with compensatory increase in parathyroid hormone (PTH); however, there have been few reported cases in peritoneal dialysis patients [5]. We present a patient with ESRD on peritoneal dialysis who developed severe symptomatic hypocalcemia and dramatic increase in PTH following denosumab therapy. We also reviewed the literature on the use of denosumab in dialysis patients and looked at additional factors that may precipitate severe hypocalcemia in them.

## 2. Case Report

A 55-year-old Caucasian woman on peritoneal dialysis for the last three years due to lupus nephritis was seen in the dialysis clinic for a routine follow-up. Her medical problems included hypertension, secondary hyperparathyroidism, and documented osteoporosis on DEXA scan (Dual energy X-ray absorptiometry) with left femoral neck BMD at 0.637 g/cm^2^; 2.9 standard deviation below peak BMD. Her medications included Amlodipine 5mg, Cinacalcet 60 mg, and Epoetin weekly injections.

She complained of fatigue, muscle cramps, and paresthesias of the hands and feet for the past week. Vital signs were stable. Physical exam was unremarkable with negative Chvostek's and Trousseau's signs. Laboratory work-up showed profound hypocalcemia with a total calcium level of 6.4 mg/dL with corrected calcium level of 6.9mg/dL (serum albumin level of 3.4 g/dL) and markedly increased serum intact PTH level (iPTH) of 2601 pg/mL (Table 1). Serum phosphate and serum alkaline phosphatase level was within normal reference range at 3.1 mg/dl and 84 U/L, respectively. Upon history taking, she was found to have received a new medication, denosumab 60mg subcutaneously, prescribed by her rheumatologist 10 days prior to the current presentation.

Before the administration of denosumab, the patient had normal serum values of calcium (9.5 mg/dL), phosphorus (3.8 mg/dL), alkaline phosphatases (96 U/L), and 25 vitamin D with slightly elevated iPTH level at 442 pg/mL. The patient was supplemented with 3,000 mg/dL of oral calcium along with oral Calcitriol 0.5mcg daily and Cinacalcet was held. The peritoneal dialysis solution was also changed from low calcium to regular calcium. All these measures resolved her symptoms with serum calcium normalizing (corrected calcium, 8.7 mg/dL) 5 days later whereas iPTH took almost 4 weeks to come back to baseline level (Table 1). Subsequently, she was managed as an out-patient with close monitoring of calcium homeostasis.

## 3. Discussion

CKD is characterized by spectrum of mineral and bone disorders (CKD-MBD) that worsen with progressive loss of kidney function and is associated with an increased risk of fragility (low trauma) fractures. Presently, there is no established effective therapy for patients having CKD-MBD with low BMD (stage 4-5D) or fragility fractures [2, 5]. Bisphosphonates, which are the longest established and most widely prescribed osteoporosis treatment class, are not recommended in patients with severe renal impairment due to their direct nephrotoxic effect, risks of adynamic bone disease, and prolonged retention in the bone [2].

Denosumab is an anti-resorptive drug which has been FDA approved since 2010 for the treatment of postmenopausal osteoporosis and recently for the prevention of skeletal-related events in patients with bone metastases [6, 7]. It is a fully human monoclonal antibody directed against the receptor activator of nuclear factor-*κβ* (RANK) ligand. Normally, binding of RANK ligand to its receptor results in activation of osteoclasts, and thus inactivating the RANK ligand with denosumab reduces osteoclasts activity and bone resorption. It is cleared by the reticuloendothelial system and treatment efficacy of denosumab is neither affected by kidney function nor it affects the kidney function [8]. When compared with bisphosphonates, it offers improved efficacy, better tolerability, and convenient administration via subcutaneous injection making it a preferable choice in patients with advanced kidney disease [9].

However, despite these benefits, denosumab has been associated with severe hypocalcemia which is usually asymptomatic but can also present in certain high-risk patients with serious manifestations, including cardiac arrhythmia and death [5, 10]. The generally recognized risk factors for hypocalcemia include osteoblastic metastases, high bone turnover states, elevated alkaline phosphatase, vitamin D deficiency, concomitant bisphosphonates use, and renal dysfunction with creatinine clearance (CrCl) of less than 30 mL/min and/or dialysis dependence [10]. A recently published meta-analysis of six observational studies [11] assessing the incidence of denosumab-associated hypocalcemia in 84 ESRD patients found that the pooled estimated incidence of hypocalcemia was as high as 42% (95% CI 29–55%, *I*^2^ = 0%). The authors cautioned against the use of this medication in ESRD patients even though there was significant increase in BMD proving its efficacy in these patients. In another study of the 85 women with postmenopausal osteoporosis treated with denosumab, 22 (25.9%) developed hypocalcemia who had significantly higher bone turnover markers at baseline (e.g., total N-terminal propeptide of type 1 procollagen, tartrate-resistant acid phosphatase 5b, etc.) suggesting that high bone turnover is potentially a risk factor in denosumab-induced hypocalcemia [12]. Our patient had ESRD, secondary hyperparathyroidism that is associated with a high bone turnover status, and was on Cinacalcet, thus posing a high cumulative risk for development of hypocalcemia on receiving denosumab.

The transient increase in PTH is expected after the drug administration which helps to maintain calcium homeostasis. This is mostly seen in patients with severe renal impairment (CrCl<30 mL/min) or on dialysis, in whom secondary hyperparathyroidism is also common, making it an expected finding. In a retrospective study performed in 14 patients with CKD stages 4–5 treated with denosumab [5], a 3.1-fold rise in the mean PTH level was observed at the time of the corrected calcium nadir (defined as the lowest observed corrected calcium level occurring in the first 90 days after receiving the medication). However, our patient had a dramatic increase in her iPTH which has been sporadically reported before. Torregrosa [13] published intense prolonged hypocalcemia and hyperparathyroidism after denosumab in a kidney transplanted patient while Martin-Gomez and colleagues [14] recently reported similar results in a hemodialysis patient. Consistent with these reported cases, this finding is likely the result of profound hypocalcemia in our patient who already had severe secondary hyperparathyroidism necessitating Cinacalcet therapy at baseline.

Denosumab-induced hypocalcemia usually lasts for few weeks and the risk has been found to be highest in the first 2 weeks [15]. However, certain case reports indicate that it can be more prolonged lasting up to 5-6 months [16]. Hyperparathyroidism correction appears to lag behind in most of the reported patients similar to our case. Treatment usually includes calcium and vitamin D supplementation, dose reduction or stoppage of hypocalcemic agents such as Cinacalcet, and dialysis using high calcium dialysate in severe, resistant cases [5, 9].

To conclude, the usage of denosumab has increased for osteoporosis. However, its use in patients with advanced chronic kidney disease requires caution since it may lead to profound hypocalcemia. There appears to be lack of widespread awareness among prescribing practitioners and communication gap with the nephrology team that may be contributing to this side effect being still prevalent. Having said that, recent meta-analysis does suggest an efficacy of denosumab in the improvement in BMD among ESRD patients on dialysis and the benefit of the drug is prevailing. We advocate careful consideration before using denosumab in dialysis patients and notifying patient's nephrologist when initiating the treatment so that close biochemical monitoring can be arranged. Certain preventive strategies including appropriate adjustment in calcium dialysate, adequate calcium and vitamin D supplementation, and avoiding concomitant use of hypocalcemic agents such as Cinacalcet can help to potentially prevent symptomatic hypocalcemia in ESRD patients treated with denosumab.

## Figures and Tables

**Table 1:** Laboratory data at baseline and after denosumab therapy.

Laboratory parameters	Before treatment	After denosumab therapy			Reference range
		Day 10	Day 15	Day 30	
Corrected Calcium (mg/dL)	9.9	6.9	8.7	9.3	8.4 – 10.2

Phosphorus (mg/dl)	3.8	3.1	3.6	3.6	2.5 – 5.0

Intact PTH (pg/ml)	442	2601	799	349	150 – 300 (Caucasian Dialysis patients)

Alkaline phosphatase (U/L)	96	84	54	49	34- 104

25-OH Vitamin D (ng/ml)	35	_	_	_	30 - 100
